# The Impact of a Specialized Team in the Management of Patients With Incisional Hernias: Experience From a Secondary Public Hospital

**DOI:** 10.7759/cureus.90966

**Published:** 2025-08-25

**Authors:** Thiago Henrique Sigoli Pereira, Gustavo Volpe, Marcos Borges, José Sebastião Dos Santos

**Affiliations:** 1 Trauma and Acute Care Surgery, Ribeirao Preto Medical School, University of Sao Paulo, Ribeirão Preto, BRA; 2 Cardiology, Ribeirao Preto Medical School, University of Sao Paulo, Ribeirão Preto, BRA; 3 Internal Medicine, Ribeirao Preto Medical School, University of Sao Paulo, Ribeirão Preto, BRA; 4 Surgery and Anatomy, Ribeirao Preto Medical School, University of Sao Paulo, Ribeirão Preto, BRA

**Keywords:** general surgery, hernia recurrence, herniorrhaphy complications, incisional hernia, secondary health center

## Abstract

Introduction: Large incisional hernia is a complex pathology, and its treatment yields better outcomes when performed by a surgical team specialized in abdominal wall management. Waiting lists are long, and many patients fail to meet the minimum criteria for surgery due to morbid obesity, poor control of comorbidities, or ongoing smoking. While waiting, patients often experience pain crises, increased hernia volume, loss of function, financial deterioration, and emergency procedures. In such contexts, surgery has high rates of mortality, infection, and hernia recurrence. Expanding the number of hospitals and trained teams able to perform high-quality, large incisional hernia repairs is a promising strategy to reduce wait times and improve outcomes.

Methods: We conducted a retrospective observational study including 87 patients who underwent incisional hernioplasty between August 2019 and December 2023 at a medium-complexity public hospital. Patients with hernia defects larger than 6 cm were included. Data were collected from medical and surgical records. Patients with primary, parastomal, or inguinal hernias were excluded. Statistical analysis was performed using Stata Statistical Software: Release 12.0 (StataCorp LLC, College Station, Texas, United States), with logistic regression applied to identify predictors of surgical site infection, hernia recurrence, and reintervention. A p-value ≤ 0.05 was considered statistically significant

Results: Among the 87 patients included, surgical site infection occurred in 22 (25.3%). In multivariate analysis, surgical site infection was independently associated with seroma (OR 3.77; 95%CI 1.37-10.34; p = 0.01) and wound dehiscence (OR 5.73; 95%CI 1.80-18.23; p = 0.003). Hernia recurrence was significantly associated with diabetes (OR 2.23; 95%CI 1.22-25.95; p = 0.026), hypothyroidism (OR 2.04; 95%CI 1.06-26.56; p = 0.041), dehiscence (OR 2.22; 95%CI 1.22-25.42; p = 0.026), and surgical site infection (OR 2.86; 95%CI 2.17-64.13; p = 0.004).

Conclusion: This study adds evidence supporting the establishment of specialized teams and targeted care for patients with incisional hernias within the Brazilian Unified Health System (SUS).

## Introduction

Incisional hernia is defined as any defect, with or without protrusion, in the area of a previous surgical incision, clinically or radiologically detectable [1]. It can be symptomatic in up to 78% of patients, with complaints of local pain, dyspepsia, incarceration, or even strangulation [2]. Incisional hernia repair occurs in 5-15% of cases under emergency conditions due to acute complications and has high morbidity and mortality rates compared to elective surgery [3]. While incisional hernias occur in 5-15% of patients undergoing midline laparotomy, in high-risk populations, this prevalence may reach 30%. The main risk factors are obesity (BMI ≥30 kg/m²) and aneurysm repair surgery [4]. Patients who develop incisional hernias have a 2.5 times higher rehospitalization rate than those who undergo the same surgeries without developing a hernia [5].

Incisional hernias present in multiple forms and are difficult to classify and standardize in treatment. An effort led by the European Hernia Society (EHS) resulted in the most widely used current classification, based on location, width, length, and prior repairs. This format addresses the main risk factors for recurrence and surgical site complications [6]. Slater et al. later introduced the concept of the “complex hernia,” categorizing patients into minor, moderate, and major levels of complexity to identify those who should be referred to specialized centers and to anticipate technically demanding procedures associated with longer operative times and higher treatment costs [7]. More recently, the EHS revisited this concept through a Delphi consensus process, incorporating eighteen variables; the presence of one or more of these factors defines a complex incisional hernia [8].

The Ventral Hernia Working Group (VHWG) developed a preoperative approach that classifies patients based on risks for surgical site events, hernia recurrence, and also guides the choice of optimal mesh [9]. Interestingly, a 5% reduction in incisional hernia incidence after laparotomy would save more than €4 million per year in France alone, in both direct and indirect costs [10]. In the United States, an estimated $6.3 billion is spent on ventral and incisional hernia repairs in hospital charges alone [11].

The main modifiable risk factors associated with higher perioperative complications and total treatment costs are class III obesity and poorly controlled diabetes (HbA1c >8%). Patients with BMI ≥40 kg/m² incur an average of $3,600 more in total costs when experiencing major perioperative complications compared to those with lower BMI [12].

Surgical site infection (SSI) is the Achilles’ heel of abdominal wall surgery. It leads to increased recurrence rates, prolonged hospital stays, reoperations, higher costs, and worse postoperative quality of life. To date, no algorithm reliably controls infection in high-risk patients [13]. Another challenging clinical scenario is the "loss of domain" hernia, in which herniated viscera become permanently housed in the hernia sac, effectively forming a secondary abdominal cavity. For these patients, preoperative computed tomography (CT) volumetric analysis is beneficial for estimating hernia and abdominal cavity volumes. If the calculated volume ratio is greater than 25%, a progressive pneumoperitoneum may be a useful strategy prior to definitive repair [14].

Patients with large incisional hernias often face significant delays between diagnosis and surgical repair due to long waitlists, active surveillance (non-operative management), or preoperative optimization needs. A waiting time of just six months is associated with higher rates of failure and increased hernia sac volume [15]. Therefore, surgical queue prioritization should follow objective criteria, such as frequent analgesic use, hernia-related quality-of-life indices, and a risk-benefit analysis regarding comorbidity optimization [16,17].

Some practices in incisional hernia repair are well established, such as fascial closure whenever possible, mesh reinforcement, and antibiotic prophylaxis [18]. However, several aspects remain under debate: use of drains, abdominal binders, component separation techniques, or prophylactic mesh placement after midline laparotomy [19,20].

Closure of large hernia defects without tension requires medial myofascial advancement via component separation. The oldest and most widespread technique is anterior separation, proposed by Albanese [21], modified by Ramirez in 1990 [22]. Since then, various adaptations have aimed to reduce surgical site complications and increase medial advancement, such as retromuscular component separation with transversus abdominis release (TAR), anterior separation preserving perforator vessels [23], and endoscopic-assisted anterior separation [24]. Although no study has definitively established the best technique, many authors advocate for TAR in complex hernias, a modification of the Rives-Stoppa approach [25]. Traditional anterior component separation is associated with more surgical site complications, whereas TAR is linked to worse quality-of-life indicators in the first postoperative year [26,27].

This study aims to identify predictive factors associated with surgical site infection, recurrence, and reintervention in patients undergoing incisional hernia repair in a public hospital setting between 2019 and 2023. It shows that reoperations and hernia recurrence are closely related to surgical site infection. Additionally, hernia recurrence is associated with preoperative diabetes. Identifying key modifiable risk factors allows for better selection of surgical candidates. Combined with standardized care from a specialized abdominal wall team, these efforts improve postoperative outcomes. This study may help fill a gap in the literature and support the development of protocols aimed at optimizing care in this complex surgery.

## Materials and methods

This was a retrospective observational study conducted at Serrana State Hospital, São Paulo, Brazil, a medium-complexity public facility equipped with intensive care units and capable of performing major abdominal surgeries. The study analyzed patients who underwent incisional hernioplasty between August 2019 and December 2023. The study was approved by the Research Ethics Committee, Hospital Das Clínicas Da Faculdade De Medicina De Ribeirão Preto (approval number: 88644025.1.0000.5440) under exemption from informed consent, given the retrospective nature of the study and the use of anonymized data. The study was conducted in accordance with the Declaration of Helsinki and Brazilian National Health Council Resolution No. 466/2012.

Inclusion and exclusion criteria

We included patients with incisional hernias measuring greater than 6 cm in diameter, classified as medium to large-volume defects. Hernia size was determined from the operative records. A total of 87 patients met the inclusion criteria. Exclusion criteria were primary hernias, parastomal hernias, and inguinal hernias, as these involve different pathophysiology and surgical approaches.

Perioperative care

All patients received antibiotic prophylaxis with cefazolin (or clindamycin if allergic) administered until one hour before skin incision. No additional standardized perioperative or postoperative protocol was employed beyond routine institutional care. 

Surgical technique

Primary fascial closure with mesh reinforcement was attempted in all cases. Four operative techniques were employed: (i) Onlay repair: mesh was placed over the anterior rectus sheath/aponeurosis, (ii) Retromuscular repair: mesh was positioned in the retrorectus plane, (iii) TAR: posterior rectus sheath was incised and the transversus abdominis muscle divided to allow mesh placement in an extended retromuscular space, (iv) Alcino Lázaro’s technique: posterior rectus sheath release was combined with mesh reinforcement, a regional adaptation commonly practiced in Brazil.

The choice of technique depended on intraoperative findings, hernia characteristics, and surgeon preference. Mesh fixation and drain placement were also determined by the operating surgeon.

Data collection

Clinical, demographic, and surgical data were retrospectively collected from the electronic medical records and operative reports. No changes were made to the perioperative or postoperative care provided to the patients. Data collected included age, sex, comorbidities, body mass index (BMI), active smoking, hernia ring size, surgical technique used, length of hospital stay, postoperative complications, and recurrence.

Statistical analysis

All statistical analyses were performed using Stata Statistical Software: Release 12.0 (StataCorp LLC, College Station, Texas, United States). Descriptive statistics were used to summarize demographic and clinical characteristics. Categorical variables were expressed as absolute numbers and percentages, and continuous variables as means and standard deviations. To assess associations between risk factors and clinical outcomes, bivariate analysis was conducted using the Student’s t-test for continuous variables and the Pearson chi-square test for categorical variables. A multivariate logistic regression model was then applied to identify independent predictors of three key outcomes: surgical site infection, hernia recurrence, and surgical intervention. The results of the logistic regression are reported as odds ratios (ORs) with 95% confidence intervals (CIs), p-values, and the corresponding Z statistics. A p-value ≤ 0.05 was considered statistically significant.

## Results

The analyzed sample showed a balanced gender distribution, with a mean age of 56.1 years. There was a predominance of patients with grade 1 obesity (n=35, 40.2%), the vast majority being non-smokers (n=75, 86.2%), and a diabetes prevalence of 23 (26.4%). The average diameter of the hernia rings was 13.3 cm, and the SSI rate was 22 (25.2%), although more than half of the patients experienced some type of surgical site occurrence (Table 1).

**Table 1:** Demographic characteristics of patients

Characteristics	Value
Gender, n (%)	
Female	43 (49.4%)
Male	44 (50.6%)
Age (years), mean (range)	56.8 (21-81)
Body Mass Index (BMI), n (%)	
Normal	16 (18.3%)
Overweight	29 (33.3%)
Obesity Grade 1	35 (40.2%)
Obesity Grade 2	7 (8%)
Active Smoking, n (%)	
Yes	12 (13.8%)
No	75 (86.2%)
Diabetes, n (%)	
Yes	23 (26.4%)
No	64 (73.5%)
Hernia Ring Size (cm), mean (range)	13.3 (6-30)
Length of Hospital Stay (days), mean (range)	4.9 (1-60)
Postoperative Follow-up Time (months), mean (range)	8.8 (1-48)
Surgical Site Ocorrences	
Yes	45 (51.7%)
No	42 (48.3%)
Surgical Site Infection	
Yes	22 (25.2%)
No	65 (74.8%)
Surgical Techniques	
Onlay	58 (66.6%)
Retromuscular	10 (11.4%)
Alcino-Lázaro	13 (14.9%)
Transversus Abdominis Release	6 (6.8%)

The analysis of the results showed that SSI was significantly associated with the formation of seromas, wound dehiscence, and the diameter of the hernia ring. Furthermore, hernia recurrence was associated with the presence of preoperative diabetes and hypothyroidism, as well as with surgical site infection and postoperative wound dehiscence. Surgical reintervention showed a strong statistical association with the presence of wound dehiscence and SSI, emphasizing the importance of strict control of these factors to optimize clinical outcomes.

Interestingly, no direct correlation was identified between obesity and worse outcomes in the analyzed sample. This finding suggests that other factors, such as glycemic control and the presence of associated comorbidities, may have a greater impact on patient prognosis. Logistic regression analysis reinforced that hernia recurrence is strongly associated with diabetes and the need for surgical intervention (Table 2).

**Table 2:** Logistic regression OR: odds ratio; CI: confidence interval; z: test statistic from Wald z-test; p: probability associated with the z statistic

Parameters	Frequency (Percentage)	OR	p-value	Z	95% CI
Surgical Site Infection		3.77	0.010	2.58	1.37-10.34
Seroma	31 (35.6%)	3.77	0.010	2.58	1.37-10.34
Wound Dehiscence	16 (18.39%)	5.73	0.003	2.96	1.80-18.23
2nd Quartile of Hernia Size	29 (33.33%)	4.57	0.017	2.39	1.31-15.93
Hernia Recurrence					
Diabetes	23 (26.4%)	5.64	0.026	2.23	1.22-25.95
Hypothyroidism	11 (12.64%)	5.32	0.041	2.04	1.06-26.56
Wound Dehiscence	16 (18.39%)	5.58	0.026	2.22	1.22-25.42
Surgical Site Infection	22 (25.28%)	11.81	0.004	2.86	2.17-64.13
Surgical Reintervention					
Wound Dehiscence	16 (18.39%)	7.61	0.006	2.72	1.76-32.82
Surgical Site Infection	22 (25.28%)	14.7	0.002	3.16	2.76-78.03

## Discussion

The study results highlight the effectiveness and safety of incisional hernia repair techniques, encompassing both traditional and modern approaches. Similar to the findings by Bittner et al. (2014) in the International Endohernia Society (IEHS) guidelines [2,3], our results reinforce that surgical technique and standardized perioperative care play a crucial role in patient outcomes.

Identifying and addressing key modifiable risk factors is crucial for improving patient outcomes. When combined with the standardized care provided by a specialized abdominal wall team, these measures can significantly reduce the incidence of postoperative complications. Our data aligns with recent research that highlights SSIs as the "Achilles heel" of abdominal wall surgery [13].

The observed recurrence rates are also consistent with those reported in large-scale retrospective analyses. However, our data did not identify some independent factors, such as obesity, smoking, age, and prior surgery, that were noted by Rhemtulla et al. (2021), who highlighted the growing burden of complex incisional hernias [5]. Additionally, Jensen et al. (2019) reported that large hernias tend to increase in size over time, emphasizing the need for timely surgical intervention [15].

Finally, as emphasized by Cano et al. (2021), optimizing surgical access through prioritization protocols and decentralization to secondary-level hospitals may expand access without compromising quality [16]. This perspective aligns with our conclusion that institutional protocols and multidisciplinary teams are key to improving surgical outcomes in public health systems.

However, this study has limitations that should be taken into consideration. One important limitation is the heterogeneity of surgical techniques employed, including Onlay, Retromuscular, Transversus Abdominis Release, and Alcino Lázaro’s repair. Although all involved primary fascial closure with mesh reinforcement and no significant differences were observed in postoperative outcomes, variability in mesh position, fixation method, and use of drains may represent potential confounding factors. Furthermore, as this is a retrospective and single-center analysis, the findings may not be fully generalizable to other institutions with different care models or patient populations. Multicenter, prospective studies with larger sample sizes will be necessary to validate and expand upon the evidence discussed here.

## Conclusions

Considering the benefits of a specialized approach for complex surgical patients, with precise indications and interventions performed at appropriate times, the data suggest a trend toward significant improvement in clinical outcomes, including lower rates of surgical site infections and hernia recurrence.

We hope to contribute to the development of preoperative optimization protocols focused on identifying and correcting risk factors, aiming to enhance surgical safety and reduce complications. Additionally, it is important to highlight the need to expand the capacity of secondary-level hospitals to perform this type of procedure with quality, thereby promoting greater access and improved effectiveness in the treatment of incisional hernias.
